# Virtual reality-assisted language learning: A follow-up review (2018–2022)

**DOI:** 10.3389/fpsyg.2023.1153642

**Published:** 2023-03-20

**Authors:** Congchao Hua, Jia Wang

**Affiliations:** ^1^School of Foreign Languages, Shenzhen Technology University, Shenzhen, China; ^2^Institute for International Students, Nanjing University, Nanjing, China

**Keywords:** trends, benefits, drawbacks, virtual reality, language learning

## Abstract

Virtual reality (VR) is considered an emerging technology in language education in a previously published review article, which reviews 26 articles on VR-assisted language learning (VRALL) published between 2015 and 2018. VR technology has been developing rapidly and receiving much more attention in language learning, especially in the post-pandemic era. Therefore, following up, this paper tracks the new trends of VRALL by reviewing 38 empirical studies published between 2018 and 2022. The main findings are: (1) the scope of research on VRALL has expanded in terms of number of studies, type of participants, research focus, language and language skill, and type of VR technology investigated; (2) more cognitive and affective benefits as well as drawbacks of VRALL have been reported than before. Implications are drawn for practitioners and researchers in the field of VRALL.

## 1. Introduction

Virtual Reality (VR), which brings authentic and immersive learning environment, is gaining increasing popularity in technology-enhanced language learning (Lin and Lan, 2015; Parmaxi, 2020; Peixoto et al., 2021). By simulating a strong illusion of presence, VR has great potential to be incorporated in language education to promote contextualized and interactive learning experiences.

Virtual Reality (VR) has been evolving rapidly since its inception as early as the 1960s. Far different from early two-dimensional (2D) text-based online virtual environments, VR tools nowadays have become much more sophisticated and interactive with three-dimensional (3D) virtual spaces and customized avatars (Lin and Lan, 2015). Despite the controversies over the definition and classification of VR (for review, see Girvan, 2018; Motejlek and Alpay, 2021), in a broad sense, it can be classified into low-immersion VR (LiVR) and high-immersion VR (HiVR; Lee and Wong, 2014; Kaplan-Rakowski and Gruber, 2019). The former refers to “a computer-generated three-dimensional virtual space experienced through standard audio-visual equipment, such as a desktop computer with a two-dimensional monitor,” while the latter refers to “a computer-generated 360° virtual space that can be perceived as being spatially realistic, due to the high immersion afforded by a head-mounted device” (Kaplan-Rakowski and Gruber, 2019, p. 552). The level of immersion mainly depends on the quality of the VR content and the gear applied for users to experience the content.

A growing number of studies have been exploring the affordances of VR-assisted language learning (VRALL). Evidence shows that VR provides simulated real-life experiences hardly accessible in traditional classroom settings, which could boost learner autonomy and engagement and reduce learning anxiety (Yeh and Lan, 2018; Parmaxi, 2020). The application of VR tools facilitates the acquisition of various language skills, such as vocabulary (Madini and Alshaikhi, 2017; Legault et al., 2019; Alfadil, 2020), speaking (Xie et al., 2019), writing (Lan et al., 2019), listening (Lan et al., 2018a,b), as well as cultural competence (Zheng et al., 2017; Chen, 2018). Moreover, teachers and learners in general report positive experience while using VR in language teaching and learning (Kaplan-Rakowski and Wojdynski, 2018; Wen, 2021).

The outbreak of COVID-19 resulted in a pressing need for VR-enhanced language learning due to the quarantine measures across the world. Since then, a surge of empirical studies have been conducted on the implementation of VR technology in language education. Parmaxi (2020) has reviewed the VRALL literature between January 2015 and September 2018, synthesizing the trends and impacts of VR as an emerging technology in language learning. To continue this line of research and delineate the development of VRALL in the post-pandemic era, this paper reviews VRALL literature between October 2018 and September 2022 with an aim to address the following two research questions:

1. What are the new trends in research on VR-assisted language learning?
2. What are the benefits and drawbacks of VR-assisted language learning?

## 2. Methodology

This review is a follow-up of Parmaxi (2020) which covers the VRALL literature between January 2015 and September 2018. In order to capture the development and trends in this field, we cover the VRALL literature between October 2018 and September 2022 in the current review.

### 2.1. Target journals

Selection of Parmaxi (2020) of target journals was mainly informed by Smith and Lafford (2009), who ranked CALL-specific and applied linguistics journals based on the quality of articles and contribution to the field. Besides, The 2018 5-year h-index and h-median metrics of Google Scholar was also included as the criteria. Under such criteria, 15 high-impact journals and conferences in the fields of computer-assisted language learning and educational technology were selected, as shown in Table 1. In order to compare our findings with those of Parmaxi (2020), we also searched VRALL-related articles from these journals and conferences. It is worth mentioning that we did not include the two conferences in Parmaxi (2020) (i.e., International Conference on Virtual System and Multimedia, and International Conference of Educational Innovation through Technology), as we failed to find resources of these two conferences in recent four years at the time of this research. We acknowledge such a limitation but this should not affect the results much since each conference yielded just one article as reported in Parmaxi (2020).

**Table 1:** Target journals and conferences selected for VRALL-related articles.

	Title of target journal/conference
1	Australasian Journal of Educational Technology
2	British Journal of Educational Technology
3	CALICO Journal
4	Computer Assisted Language Learning
5	Computers & Education
6	Educational Technology Research and Development
7	IEEE Transactions on Learning Technologies
8	International Conference on Learning Analytics and Knowledge
9	International Journal of Computer-Supported Collaborative Learning
10	Journal of Computer Assisted Learning
11	Journal of Educational Technology & Society
12	Language, Learning & Technology
13	ReCALL
14	The International Review of Research in Open and Distributed Learning
15	The Internet and Higher Education

### 2.2. Search terms

We went to the homepages of the target journals listed above and searched all the issues published between October 2018 and September 2022. In each issue, we manually identified each paper by examining the title, abstract, and keywords with terms related to “virtual reality” and “language learning,” which were covered in Parmaxi (2020). For “virtual reality,” we also securitize related terms such as “virtual exchange,” “virtual communication,” “virtual world,” “immersive environment,” “virtual environment,” and “virtual classroom.” For “language learning,” we also searched similar terms such as “second language (L2) learning,” “heritage language learning,” “computer-assisted language learning,” “technology-enhanced language learning,” “language teaching,” “language education,” “language classroom,” and “language courses.” Apart from these general terms, we also added terms on specific language skills including “vocabulary,” “writing,” “reading,” “listening,” “speaking,” “speech,” and “pronunciation.”

### 2.3. Inclusion criteria

The following inclusion criteria were applied in the collection of articles for review.

1. Adopting VR technology in language learning. Both high-immersion VR and low-immersion VR were accepted. As for language learning, L1 acquisition and L2 or foreign language learning were both included.
2. Employing empirical research methods. Articles reporting research with quantitative, qualitative, or mixed methods were included. Theoretical articles and review articles were excluded.

### 2.4. Screening and search results

The initial search yielded 114 articles in total. Based on the above inclusion criteria, the two authors made further screening separately and agreed to include those articles that explicitly stating the use of the VR technology (both low-immersion and high-immersion VR). Therefore, those that simply employed game-assisted or mobile-assisted approaches were not included. In the end, 38 articles remained for the current review. The two authors double checked these articles and achieved consensus. The number of articles eventually selected from each target journal and conference is shown in Table 2.

**Table 2:** Number of articles selected from each target journal/conference.

	Title of target journal/conference	No. of articles
1	Australasian Journal of Educational Technology	1
2	British Journal of Educational Technology	4
3	CALICO Journal	1
4	Computer Assisted Language Learning	7
5	Computers & Education	9
6	Educational Technology Research and Development	4
7	IEEE Transactions on Learning Technologies	1
8	International Conference on Learning Analytics and Knowledge	1
9	International Journal of Computer-Supported Collaborative Learning	0
10	Journal of Computer Assisted Learning	3
11	Journal of Educational Technology & Society	3
12	Language Learning & Technology	3
13	ReCALL	0
14	The International Review of Research in Open and Distributed Learning	1
15	The Internet and Higher Education	0

### 2.5. Information retrieval

Each of the 38 articles was scrutinized for key information by one of the authors. The following key information was retrieved and summarized: background information of the participants, language, and language skill, research focus, VR technology, research method, duration, and major findings.

## 3. Results

To address the first research question, the 38 articles were analyzed by year of publication, participants, research focus, language, language skill, research method, VR technology used, and duration of intervention/observation to identify the new trends in 2018–2022. To answer the second research question, the major findings of the 38 studies were summarized and categorized into benefits and drawbacks of VRALL.

### 3.1. New trends in research on VRALL

#### 3.1.1. Year of publication

**Figure 1:**
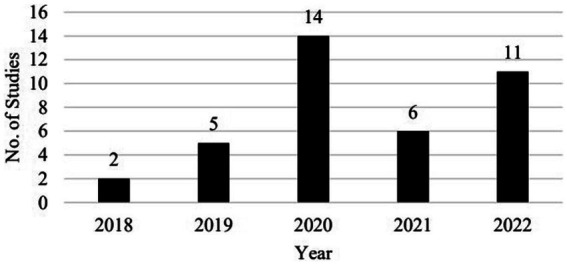
Breakdown of the studies by year of publication.

Over the 4 years (i.e., October 2018–September 2022), the number of studies published has been increasing, with the largest number of publications in 2020. It is noteworthy that we only covered part of the years of 2018 and 2022, which means that there could be more publications in these 2 years. Figure 1 is a breakdown of the 38 studies by year of publication.

#### 3.1.2. Participants

The 38 studies vary greatly by scale. The number of participants range from as few as 4 (Lan et al., 2018a,b) to as many as 274 (Chen and Hsu, 2020). Most studies (29) had 20–80 participants, a few (2) had fewer than 20, and some others (7) had more than 80.

**Figure 2:**
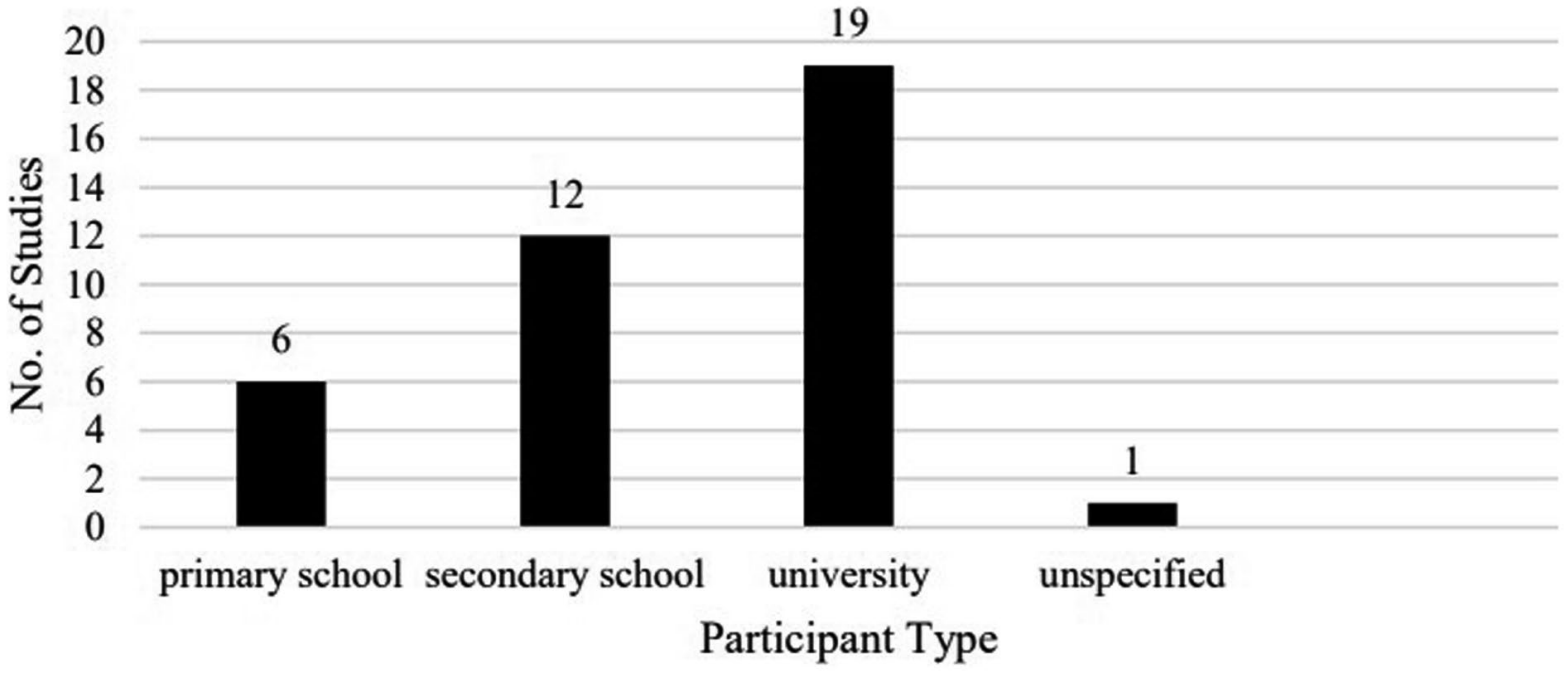
Breakdown of the studies by participant type.

Concerning the type of participants, of the 38 studies, one had unspecified participants (Rho et al., 2020), 18 investigated young learners at primary and secondary schools, and 19 examined university students (Figure 2). Table 3 lists the studies by participant type. It is worth noting that Li et al. (2020) investigated both young and adult learners aged 15–20 at secondary and university levels. In addition, two studies zoomed in on minority learners at school. Specifically, Lan et al. (2018a,b) investigated special needs students with autism at a primary school, and Ouyang et al. (2020) focused on low-achieving English learners at a junior high school.

**Table 3:** List of studies by participant type.

Young learners	Primary school students	Chen (2020), Chen Y.T. et al. (2022), Danaei et al. (2020), Lan et al. (2018a,b), Wen (2021), Yang et al. (2021)
	Secondary school students	Alfadil (2020), Chen et al. (2019), Chen et al. (2020), Chen M. P. et al. (2022), Chien et al. (2020), Huang et al. (2020), Lan et al. (2019), Li et al. (2020)*, Ouyang et al. (2020), Tai (2022), Tai et al. (2022), and Van Ginkel et al. (2020)
Adult learners	University students	Bacca-Acosta et al. (2022), Barrett et al. (2021), Chen and Hsu (2020), Chin and Wang (2021), DeWitt et al. (2022), Ebadi and Ebadijalal (2022), Fu et al. (2019), Fuhrman et al. (2020), Hsu (2022), Lee and Park (2020), Li et al. (2020)*, Liaw (2019), Ng et al. (2022), Raua et al. (2018), Shadiev et al. (2020), Shadiev et al. (2021), Teo et al. (2022), Thrasher (2022), Wang et al. (2021), Yeh and Tseng (2020)
Unspecified		Rho et al. (2020)

* Li et al. (2020) investigated students at both secondary and university levels.

#### 3.1.3. Research focus

**Figure 3:**
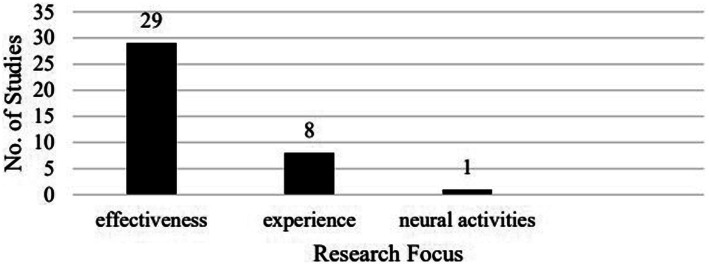
Breakdown of the studies by research focus.

The 38 studies can be divided into three groups according to research focus: assessing the effectiveness of VRALL (29), exploring learners’ experiences with VR (8; i.e., how they made use of VR for language learning), and examining neural activities during VRALL (1; Figure 3). Table 4 lists the studies by research focus.

**Table 4:** List of studies by research focus.

Assessing effectiveness of VRALL	Alfadil (2020), Chen (2020), Chen and Hsu (2020), Chen et al. (2020), Chen M. P. et al. (2022), Chen Y.T. et al. (2022), Chien et al. (2020), Chin and Wang (2021), Danaei et al. (2020), DeWitt et al. (2022), Ebadi and Ebadijalal (2022), Fu et al. (2019), Fuhrman et al. (2020), Huang et al. (2020), Lan et al. (2019), Lan et al. (2018a,b), Li et al. (2020), Liaw (2019), Ng et al. (2022), Ouyang et al. (2020), Raua et al. (2018), Rho et al. (2020), Tai (2022), Tai et al. (2022), Thrasher (2022), Van Ginkel et al. (2020), Wen (2021), Yang et al. (2021), and Yeh and Tseng (2020)
Exploring experiences in VRALL	Bacca-Acosta et al. (2022), Barrett et al. (2020), Chen et al. (2019), Lee and Park (2020), Shadiev et al. (2020), Shadiev et al. (2021), Teo et al. (2022), Wang et al. (2021)
Examining neural activities during VRALL	Hsu (2022)

#### 3.1.4. Language and language skill

The languages investigated in the 38 studies include both first language (L1) and second or foreign language (L2). Here L2 covers languages being learned in a second language environment and languages being learned in a foreign language environment, but not languages being learned as an additional or third language (L3), as none of the 38 studies claims that their focus is L3 learning.

Of the 38 studies, 28 investigated L2 learning, and 10 investigated L1 learning. Among the L2 studies, 19 examined L2 English, four L2 Chinese, two both L2 Chinese and L2 O’zbektili, and the rest three, respectively, examined L2 Finish, L2 French, and New Zealand Sign Language. Among the 10 L1 studies, eight focused on L1 Chinese (including Mandarin and Cantonese), and the other two, respectively, focused on L1 Dutch and L1 Persian. It is noteworthy that while 27 of the L2 studies assessed the application of VR in the learning of language for general use, Teo et al. (2022) examined the use of VR in the learning of language for specific purposes (L2 medical English).

**Figure 4:**
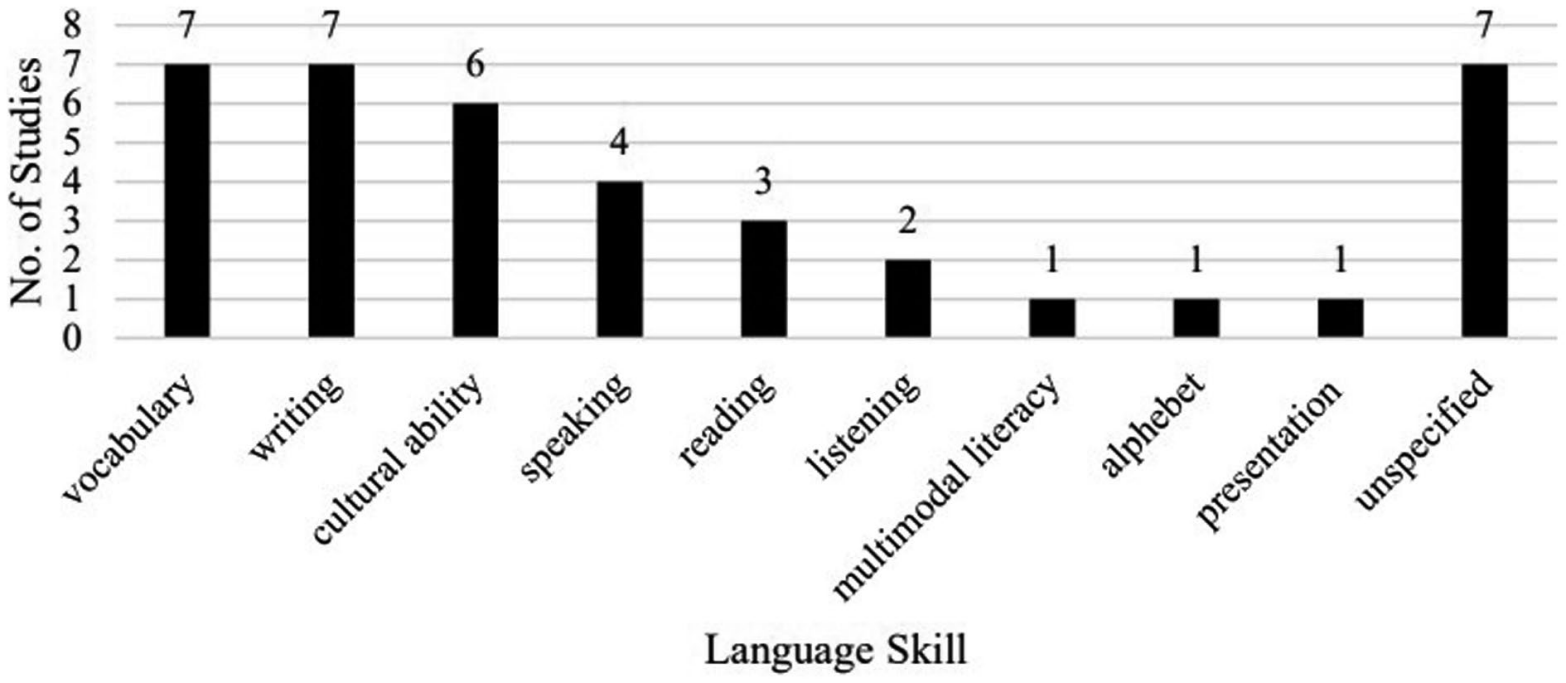
Breakdown of studies by language skill ^*^ Teo et al. (2022) investigated both listening and reading.

Among the language skills (Figure 4), the most investigated is vocabulary (7), writing (7), cultural ability covering (cross)cultural awareness, cultural knowledge, and cross-cultural communication competence (6), and speaking (4). A few investigated reading (3), listening (2), alphabet (1), multimodal literacy (1), and presentation skills (1).

Table 5 shows the list of studies by language and language skills.

**Table 5:** List of studies by language and language skill.

L1	L2	Skill	Studies
Chinese	English	Vocabulary	Chen M. P. et al. (2022), Tai et al. (2022)
		Writing	Barrett et al. (2021), Fu et al. (2019)
		Listening	Tai (2022)
		Speaking	Chien et al. (2020), Hsu (2022)
		Multimodal literacy	Yeh and Tseng (2020)
		Unspecified	Chen (2020), Chen and Hsu (2020), Chen et al. (2019), Liaw (2019), Ouyang et al. (2020)
Spanish	English	Writing	Chen et al. (2020)
Persian	English	Speaking	Ebadi and Ebadijalal (2022)
Arabic	English	Vocabulary	Alfadil (2020)
Mixed	English	Listening & reading	Teo et al. (2022)
		Unspecified	Lee and Park (2020)
Unspecified	English	Vocabulary	Bacca-Acosta et al. (2022)
English	Chinese	Unspecified	Wang et al. (2021)
Malaysian	Chinese	Cultural ability	DeWitt et al. (2022)
O’zbektili	Chinese	Cultural ability	Shadiev et al. (2020, 2021)
Mixed	Chinese	Writing	Lan et al. (2019)
		Vocabulary	Wen (2021)
Chinese	O’zbektili	Cultural ability	Shadiev et al. (2020, 2021)
Hebrew	Finnish	Vocabulary	Fuhrman et al. (2020)
unspecified	French	Speaking	Thrasher (2022)
Unspecified	New Zealand sign language	Alphabet	Rho et al. (2020)
Chinese		Writing	Chen Y.T. et al. (2022), Huang et al. (2020), Yang et al. (2021)
		Cultural ability	Chin and Wang (2021), Li et al. (2020), Ng et al. (2022)
		Reading	Raua et al. (2018)
		Vocabulary	Lan et al. (2018a,b)
Dutch		Presentation	Van Ginkel et al. (2020)
Persian		Reading	Danaei et al. (2020)

#### 3.1.5. VR technology investigated

**Figure 5:**
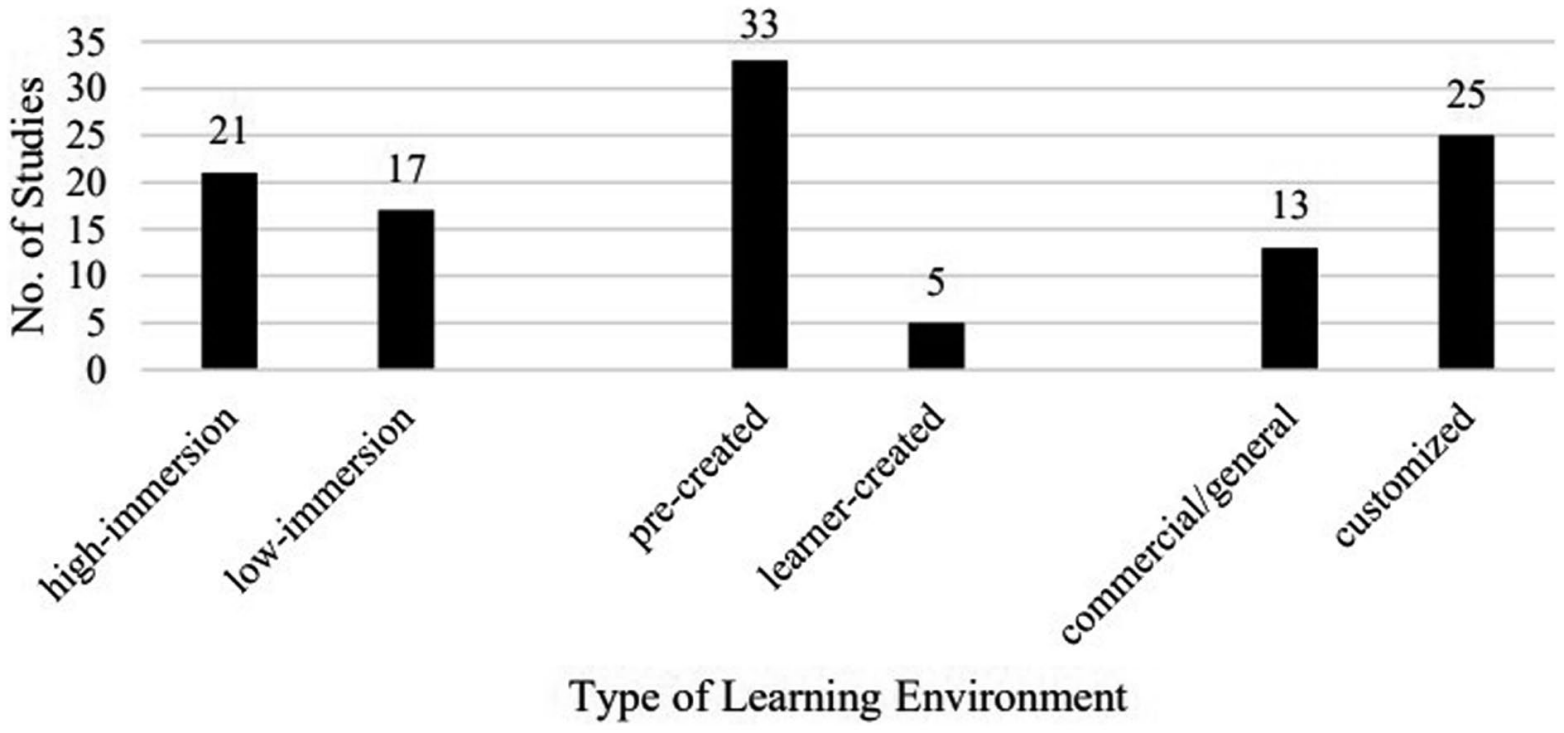
Breakdown of studies by type of VR environment.

Various types of VR technology were investigated in the 38 studies (Figure 5). Twenty-one studies report on high-immersion VR technology, and 17 low-immersion VR technology. In addition, five of the 38 studies report on VR learning platforms involving learners’ contributions such as uploading their own 360 videos to form part of the VR learning environment (Shadiev et al., 2020, 2021). Moreover, both commercial/general platforms (13) and customized, more specific platforms (25) were investigated.

The commercial or general ones include Second Life (5), vTime (2), Mondly (2), House of Languages (1), 7Scenes (1), Google Earth VR (1), Google expeditions VR (1), iMap (1), and Traveling around the United States of America (1).

The customized platforms were of various kinds and they were created by the researcher/teacher to cater to the specific objectives of the courses or language skills investigated. Among the 25 studies, nine adopted the spherical video virtual reality (SVVR) technology to create a high-immersion learning environment, such as the Virtual Reality New Zealand Sign Language application (Rho et al., 2020) and the Virtual Reality Learning Environment (Ouyang et al., 2020), and 16 adopted other technologies for a low-immersion learning environment such as the ARC&S game in Wen (2021).

Table 6 lists the studies investigating high-immersion, learner-created, and commercial/general VR environments.

**Table 6:** List of studies investigating high-immersion, learner-created, and commercial/general VR Environments.

Type of VR environment	Studies	
High-Immersion (21 studies)	Alfadil (2020), Bacca-Acosta et al. (2022), Barrett et al. (2021), Chen and Hsu (2020), Chen Y.T. et al. (2022), Chien et al., 2020, DeWitt et al. (2022), Fuhrman et al. (2020), Huang et al. (2020), Lan et al. (2019), Liaw (2019), Ouyang et al. (2020), Raua et al. (2018), Rho et al. (2020), Shadiev et al. (2020, 2021), Tai (2022), Tai et al. (2022), Thrasher (2022), Wang et al. (2021), Yang et al. (2021)	
Learner-created (Five studies)	DeWitt et al. (2022), Lee and Park (2020), Liaw (2019), Shadiev et al. (2020), Shadiev et al. (2021), Thrasher (2022), Wen (2021), Yeh and Tseng (2020)	
Commercial/General (13 studies)	Second Life	Lan et al. (2019), Lan et al. (2018a,b), Wang et al. (2021)
	Mondly	Tai (2022), Tai et al. (2022)
	vTime XR	Liaw (2019), Thrasher (2022)
	Google Earth VR	Chen et al. (2020)
	Google Expeditions VR	Ebadi and Ebadijalal (2022)
	7Scenes	Lee and Park (2020)
	House of Languages	Alfadil (2020)
	Traveling around the United States of America	Chen et al. M.P. (2022)
	iMap	Chen et al. (2019)

#### 3.1.6. Research method

**Figure 6:**
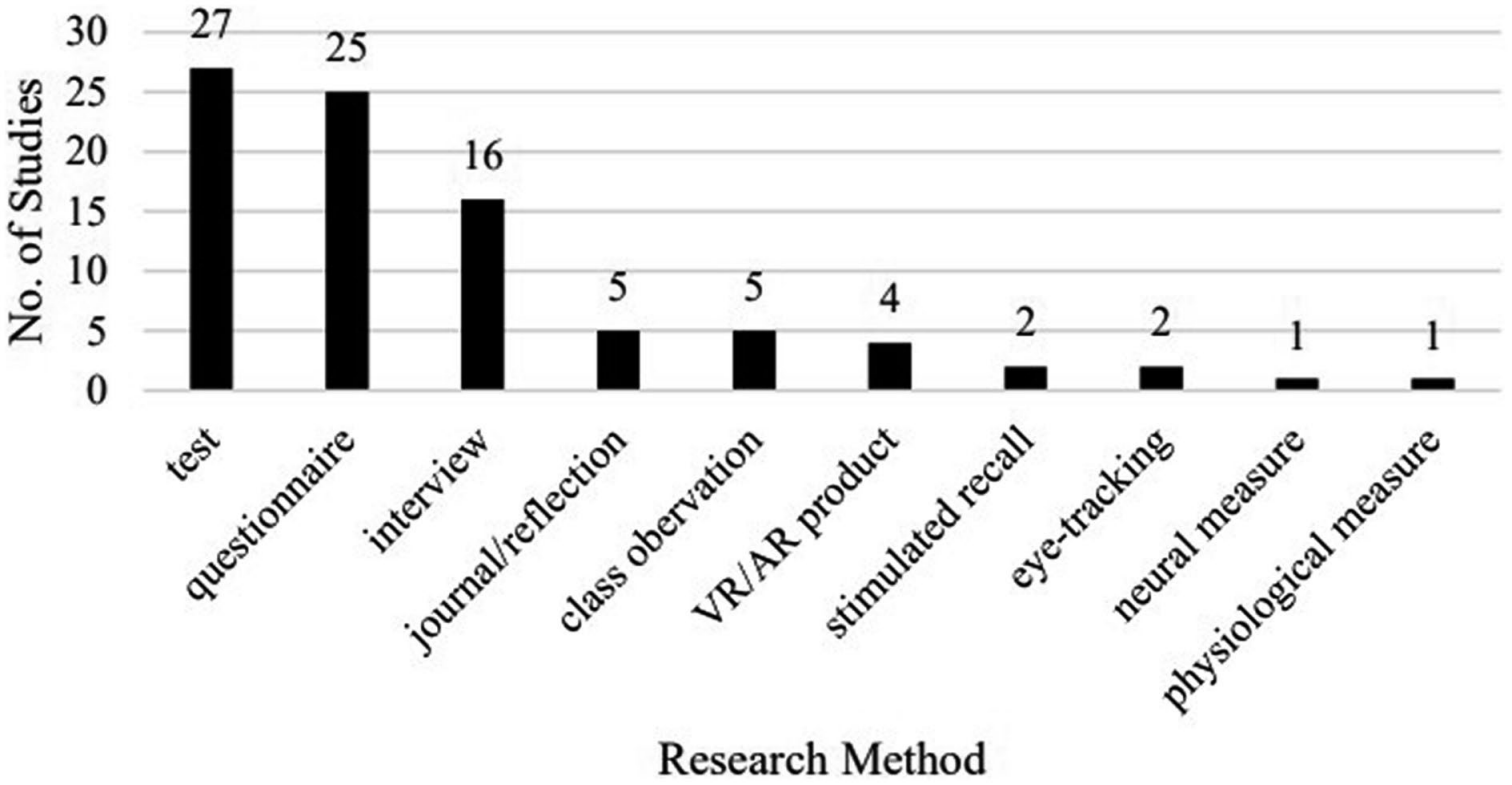
Breakdown of studies by research method.

A vast majority (36) of the studies adopted a mixed-methods approach, with just two exceptions using a single research method (i.e., Raua et al., 2018; Fuhrman et al., 2020). The research methods adopted in the 38 studies (Figure 6) cover experiment and test (27), questionnaire (25), interview (16), journal and self-reflection (5), in-class observation and video-recorded class observation (5), analysis of VR product (4), stimulated recall (2), eye-tracking (2), neural measurement (1), and physiological measurement (1).

Among the 27 studies with the experiment and test design, 20 had a pre-posttest design, among which only two had delayed posttests (i.e., Tai, 2022; Tai et al., 2022).

#### 3.1.7. Duration

**Figure 7:**
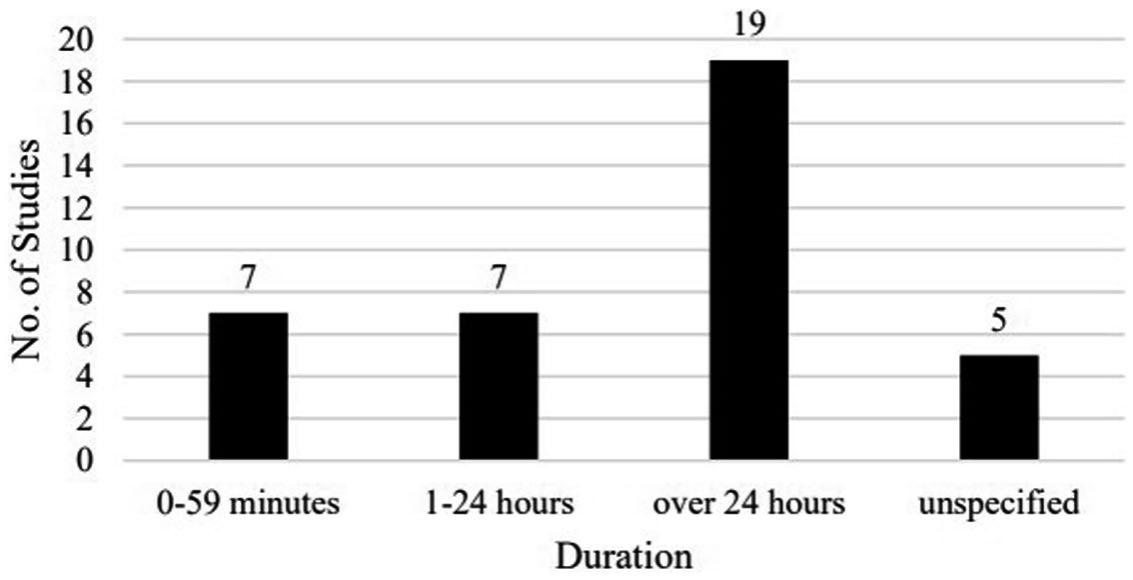
Breakdown of the studies by duration.

The duration of the intervention/observation in the studies spans from 25–35 min (Tai, 2022; Tai et al., 2022) to 1,800 min (Fu et al., 2019; Figure 7). In seven studies, the intervention/observation lasted less than 1 h, and in another seven, it lasted 1–24 h, mostly 60–90 min. In 19 studies, the whole research process lasted over 24 h, mostly in weeks, even months. However, in 17 of these 19 studies, the exact duration of intervention/observation is unclear, as it would be impossible for the research to go on 24 h non-stop during the weeks or months. In the rest five studies, the duration is not specified.

### 3.2. Benefits and drawbacks of VRALL

To answer the second research question, we summarized and categorized the major findings of the 38 studies. These include both the positive and negative effects of VR technology on language learning.

#### 3.2.1. Benefits

The benefits of VRALL reported in the studies can be classified into cognitive benefits and affective benefits (Table 7). Cognitive benefits are positive effects on language learning, whereas affective benefits refer to positive effects on the participants’ psychological and emotional aspects, which may, in turn, promote language learning.

**Table 7:** Benefits of VRALL.

	Cognitive benefits	Studies
1	Provided language learning opportunities	Lee and Park (2020), Wang et al. (2021)
2	Improved writing skills	Chen et al. (2020), Chen Y.T. et al. (2022), Fu et al. (2019), Huang et al. (2020), Lan et al. (2019), Yang et al. (2021)
3	Improved vocabulary learning	Alfadil (2020), Fuhrman et al. (2020), Tai et al. (2022)
4	Improved speaking skills	Chien et al. (2020), Ebadi and Ebadijalal (2022), Thrasher (2022)
5	Improved reading skills	Danaei et al. (2020), Teo et al. (2022)
6	Improved listening skills	Tai (2022), Teo et al. (2022)
7	Enhanced overall language learning	Chen and Hsu (2020), Ebadi and Ebadijalal (2022), Lan et al. (2018a,b), Ouyang et al. (2020)
8	Enhanced cultural competencies	Chin and Wang (2021), DeWitt et al. (2022), Ebadi and Ebadijalal (2022), Li et al. (2020), Liaw (2019), Ng et al. (2022), Shadiev et al. (2021), Shadiev et al. (2020)
9	Promoted multimodal literacy	Yeh and Tseng (2020)
10	Promoted presentation skills	Van Ginkel et al. (2020)
11	Helped with memory retention	Rho et al. (2020)
12	Reduced cognitive load	Huang et al. (2020)
13	Enhanced critical thinking	Chien et al. (2020)
Affective benefits		Studies
1	Promoted task engagement	Chen and Hsu (2020), Chen et al. (2020), Chen Y.T. et al. (2022), Chin and Wang (2021), Tai (2022), Tai et al. (2022), Wen (2021)
2	Enhanced motivation	Chen (2020), Chen and Hsu (2020), Chen M.P. et al. (2022), Chien et al. (2020), Ebadi and Ebadijalal (2022), Lan et al. (2019), Li et al. (2020), Liaw (2019), Rho et al. (2020), Tai (2022), Tai et al. (2022)
3	Elicited positive attitudes and emotions	Chen (2020), Chen et al. (2020), Chen M.P. et al. (2022), DeWitt et al. (2022), Fu et al. (2019), Lee and Park (2020), Liaw (2019), Ouyang et al. (2020), Shadiev et al. (2020), Shadiev et al. (2021), Tai et al. (2022), Yang et al. (2021)
4	Promoted willingness to communicate	Ebadi and Ebadijalal (2022)
5	Promoted confidence	Ebadi and Ebadijalal (2022), Huang et al. (2020), Rho et al. (2020)
6	Reduced foreign language anxiety	Chien et al. (2020), Thrasher (2022)
7	Reduced psychological distance between students and teacher	Teo et al. (2022)

It is not surprising that the 29 studies assessing effectiveness of VRALL generally yielded positive results: VR technology benefited the learning of all languages and all language skills by both young and adult learners. In addition, VR technology also has the potential of boosting learners’ cognitive abilities during language learning, as it promoted memory retention, reduced cognitive load, and improved critical thinking. Thirteen of the 29 studies also report learners’ positive perceptions of VRALL.

Likewise, studies examining language learners’ experiences in VRALL also generally report positive findings. These findings are mainly about the affective benefits of VR, including promoting positive attitudes and emotions (such as likes, interest, enthusiasm, enjoyment, satisfaction, etc.) and translating them into positive actions (task engagement), enhancing positive psychology (such as motivation, confidence, and willingness to communicate), mitigating negative psychology (such as foreign language anxiety), and improving teacher-student relationship.

#### 3.2.2. Drawbacks

Although research findings are overwhelmingly positive and optimistic, six studies did identify drawbacks of VRALL (Table 8).

**Table 8:** Drawbacks of VRALL.

Drawbacks		Studies
	Posed technical challenges during learning	Lee and Park (2020)
2	Provided inauthentic learning materials	Lee and Park (2020)
3	Could be time-consuming	Chen et al. (2020)
4	Could distract learners from their task	Chen et al. (2020)
5	Lowered learners’ attention and level of thinking	Hsu (2022)
6	Slowed down speed in answering questions while not improving accuracy	Raua et al. (2018)
7	Reduced confidence	Chen et al. (2019)
8	Elicited mixed feelings	Chen et al. (2020), Ouyang et al. (2020)

The drawbacks in Table 8 are much smaller in number than the benefits listed in Table 7. These drawbacks include technical issues not new to technology-enhanced language classes: unstable internet connection, and huge storage size of the learning materials (Lee and Park, 2020). Another drawback is related to VR learning materials, some of which were inauthentic (Lee and Park, 2020). Still some learners felt that using VR in the language class was time-consuming and even distracting (Chen et al., 2020). In addition, it was found that VR environment might lower learners’ attention and meditation during communication (Hsu, 2022), and slow down their speed in answering questions while not improving their accuracy (Raua et al., 2018). Moreover, concerning caption, a feature incorporated in VR language learning platforms, findings were mixed. According to Ng et al. (2022), captions divert learners’ attention during learning and thus reduce the effectiveness of VRALL. Chen et al. (2019) report that while female learners performed comparably with or without captions in a VR language learning platform, male learners performed worse with captions, and that learners were less confident in the captioned condition. In the similar vein, Ouyang et al. (2020) report learners’ mixed attitudes to captions: while some thought they were helpful, others felt that they were interfering.

## 4. Discussion

After reviewing the empirical studies on VRALL published in 2018–2022, some comparisons can be made with Parmaxi (2020) to delineate the new development and new findings in this research area.

### 4.1. New trends of research on VRALL

The obvious trends emerging from the reviewed studies are the expansion of the scope of research in terms of number of studies, type of participants, research focus, language and language skill, and type of technology investigated.

#### 4.1.1. Increase in the number of publications on VRALL

The number of publications on VRALL increased sharply after the outbreak of the pandemic in early 2020, when online teaching became a common practice to coexist or even replace face-to-face offline teaching. This increase in the number of research will probably continue given the momentum in the development and application of VR technologies in language education.

#### 4.1.2. Expansion of VRALL from universities to secondary and primary schools

While university students remained the largest group of language learners investigated (19/38) as in Parmaxi (2020) (12/26), an increasing number of studies have turned to secondary school students (12/38) and primary school pupils (6/38). It is worth noting that Parmaxi (2020) reports only one out of 26 studies investigating secondary school students, but here we found 12 out of 38. Considering the greater difficulty in accessing young learners than accessing adult learners because of ethical and practical issues, this is indeed a dramatic increase. This suggests that in the past several years, VR expanded its influence in school settings from adult learners to young learners.

#### 4.1.3. Expansion of research focus

While the majority of studies (29/38) examined the effectiveness of VRALL, the emerging trend is that quite some studies (8/38) have turned to explore language learners’ experiences in VRALL, which was the focus of only one out of the 26 studies in Parmaxi (2020). In addition, we found one study examining learners’ neural activities during VRALL. These changes indicate the expansion of the scope of research on VRALL and the emergence of inter-disciplinary research on this topic.

#### 4.1.4. Increase in language and language skills investigated

Like reported in Parmaxi (2020), English is by far the most investigated language, mostly English as a second or foreign language (18/38) or English for specific purposes (1/38). Different from Parmaxi (2020), more studies in 2018–2022 investigated Chinese (14/38), which ranks as the second most researched language in this review. Among these 14 studies, six are about L2 Chinese learning and eight about L1 Chinese learning. In addition, there is a larger variety of languages investigated. Apart from English and Chinese, a few studies focused on O’zbektili (2/38), Finnish (1/38), French (1/38), Dutch (1/38), Persian (1/38), and even New Zealand Sign Language (1/38), while Parmaxi (2020) only reports studies on Spanish, Japanese, and French apart from English and Chinese.

Our findings differ greatly from Parmaxi (2020) in terms of the language skills investigated in the empirical studies on VRALL. While Parmaxi (2020) found that speaking (8/26) was the most researched language skill, listening (2/26), and vocabulary (1/26) were far less investigated, and writing and reading were not covered in the studies reviewed, we found that vocabulary (7/38), writing (7/38), and speaking (4/38) are the top three language skills investigated, followed by reading (3/38) and listening (2/38). In addition, like Parmaxi (2020), who reports quite a few studies (5/26) examining VR in developing communicative skills, we also found some studies with a similar focus (6/38). Moreover, a few of the studies we have reviewed here focused on alphabet (1/38), multimodal literacy (1/38), and presentation skills (1/38). These changes indicate that more languages and language skills have become the focus of research on VRALL.

#### 4.1.5. Increase in application of high-immersion, interactive, and customized VR technologies

Although Parmaxi (2020) did not classify the empirical studies under review into high- and low-immersion technologies, from the description in the review most of the studies were about low-immersion technologies. In comparison, we found over half of the reviewed studies (21/38) investigating high-immersion VR technologies. Another trend is that there have appeared quite some studies (5/38) on VR technologies that learners could use to create and share their own works during language learning. In addition, compared with Parmaxi (2020), who reports five out of 26 studies examining customized platforms, we found that there have been more studies on customized VR platforms (25/38) than commercial or general platforms (13/38). Of the commercial platforms, Second Life still remained the most researched, but the number of studies is much smaller than in Parmaxi (2020) (5/38 vs. 15/26). Moreover, we also found that the spherical video virtual reality technology has attracted increasing attention from researchers in the past few years. In brief, VR technologies in language learning reported in the studies are becoming more immersive, more interactive, and more customized.

#### 4.1.6. Large variety of research methods

While Parmaxi (2020) does not specifically list all research methods adopted in the reviewed studies, the research methods in the studies in our review vary vastly, from the most common experiment to the most up-to-date eye-tracking, neural measurement, and physiological measurement. Most of the studies (36/38) are of a mixed-methods design, and many of the experimental studies (13/29) also examined learners’ perceptions. What is worth mentioning is that only two of the 27 experimental studies adopted a pre-, post-, delayed post-test design, showing a general neglect of the long-term effect of VRALL.

#### 4.1.7. Increase of research with short duration

We found a wider span of duration than Parmaxi (2020). Parmaxi (2020) reports that only one study out of 26 had a duration of less than 5 weeks and two had a duration longer than 16 weeks, the shortest duration we found is 25–35 min, and the longest 18 weeks (100 min per week). Here we would like to emphasize that while Parmaxi (2020) grouped the studies according to number of tasks or sessions, of which the specific durations were unclear, we grouped the studies according to specific minutes if the information was available. Only when there was no specific information about the length of each session did we mark the intervention/observation in weeks or months. In this way, we got quite some studies (14/38) with a very short duration of 25–90 min, probably shorter than the duration of 5 weeks in Parmaxi (2020), and a few studies (4/38) with a duration longer than 16 weeks. This finding suggests that most of the new studies in the past few years were of a shorter duration than before, and that there is still a lack of studies with long durations.

In short, our review of the empirical studies in 2018–2022 revealed increases in the scope and variety of research in the field of VRALL.

### 4.2. Benefits and drawbacks of VRALL

With the improvement of VR technologies and increased application of such technologies in language learning, empirical research has yielded more findings in 2018–2022 than before.

As reported in Parmaxi (2020), cognitively VR promoted the learning of different language skills, including speaking, listening, vocabulary, cultural knowledge, communicative competence, and critical thinking; affectively VR improved learners’ motivation and task engagement and reduced foreign language anxiety. Apart from these benefits, we also found additional benefits of VRALL. In addition to the language skills mentioned above, empirical studies in our review also report VR improving reading and writing skills, multimodal literacy, and presentation skills. Moreover, VR also contributed to the other cognitive aspects of learning in that it reduced cognitive load and enhanced memory retention. Additional affective benefits in this review include promoting learners’ confidence and willingness to communicate and establishing rapport between teacher and learners.

Just as more benefits have been reported, the number of reported drawbacks has also risen. While the drawbacks of unstable technical conditions and wasting time in Parmaxi (2020) still existed, we found no mentioning of lack of multimodal resources or negative attitudes to virtual world anonymity as in Parmaxi (2020). Probably, this is because more multimodal resources were developed in recent years and teachers could customize VR learning platforms to better meet their students’ needs. Consequently, the learners in the reviewed studies might be more used to the VR technology in their learning and had more positive attitudes.

However, we did find some new drawbacks reported in the studies. While there is no criticism of lack of multimodal materials, some report inauthentic learning materials provided on VR platforms (e.g., Lee and Park, 2020). This suggests that attention has been shifted from the quantity to the quality of VRALL materials. In addition, some report that the application of VR technology could be distracting and lower learners’ attention and level of thinking (Chen et al., 2020), and that VR had no advantage over other technologies such as augmented reality (AR) and liquid crystal display (LCD), as learners were not more accurate but slower in answering questions when using VR (Raua et al., 2018). This is consistent with the drawbacks that VR is distracting and time-consuming. While using VR, language learners have to pay attention to information from more channels, thus reducing their learning efficiency. Furthermore, while many report that VR could promote confidence, Chen et al. (2019) claim that captions in VR tended to reduce learners’ confidence. Both Chen et al. (2019) and Ouyang et al. (2020) report learners’ mixed attitudes toward the captions in VR, with both positive and negative feelings. This discrepancy is not surprising given the variety of VR platforms and materials as well as individual differences between language learners.

In summary, research findings in 2018–2022 suggest more positive than negative effects of VR on language learning. Learners’ affects in learning with VR merit more, specific investigations.

### 4.3. Practical implications

Based on the review above, some practical implications can be drawn for practitioners and researchers.

#### 4.3.1. Implications for practitioners

This review has summarized the multiple benefits of VRALL, suggesting that VR is a promising technology to facilitate language learning. Therefore, it is advisable for language teachers to improve their competency in using VR technology to best incorporate it in their teaching. In the meantime, VR language learning platform and material developers should try to make VR platforms more user-friendly to save time and mitigate distraction during learning and improve the authenticity of VR materials. In addition, as research findings suggest the influence of individual differences such as gender (e.g., Chen et al., 2019) and language proficiency (e.g., Ouyang et al., 2020) on the effectiveness of VRALL, VR platform and material developers need also take the characteristics of their target learners into consideration to fully exert the potential of VR technology in language learning and avoid its drawbacks.

Moreover, as immersion is the major predictor of acceptability of VR technology by language learners (Barrett et al., 2021), the degree of immersion of VR platforms should be enhanced to better engaged learners in learning activities. This would not be difficult to achieve, as the increasing flexibility of the VR technology has allowed for the creation of more customized VR language learning platforms, and the price of VR hardware such as head mounted devices will continue to decrease with the maturity of the VR technology.

Furthermore, VR language learning platforms should be developed to cover more languages and language courses and to satisfy the needs of a larger variety of language learners. For instance, there could be more platforms incorporating VR into English for academic purpose courses and English for specific purpose courses, and more VR platforms for young language learners, special needs language learners, and language learners other than students.

#### 4.3.2. Implications for researchers

To begin with, more research on VRALL is needed for a larger variety of language learning settings and a wider range of language learners. Our review has shown that most research on VRALL was conducted in school settings, especially universities. Considering the proven and potential benefits of VRALL, it is sensible to examine the application of VR in various language learning settings, including language training centers and autonomous language learning settings. Moreover, it is also of practical and theoretical values to investigate the impact of VR technology on a variety of language learners other than university students and secondary school students. Such research may help identify the universal mechanism behind the effectiveness of VRALL and eventually optimize its implementation.

Another implication for researchers is that more languages need to be covered to ascertain the effects of VRALL. We found that although the number of languages being investigated has increased, it is still small. A majority of the studies investigated the impacts of VR on English learning and Chinese learning. Apart from these two influential languages, only a handful of other languages have been investigated. Moreover, besides L2 learning, it is also necessary to explore the effects of VR on L3 and additional language learning, especially in multilingual contexts. Research on more languages may uncover the universality and specificness of VRALL, which, in turn, may serve as reference for designing VR language learning platform and materials and VR-assisted language classes.

Furthermore, more research is needed to better understand learner experiences in VRALL and the impacts of individual differences on VRALL. Apart from how learners perceive and evaluate the language learning experience with VR, we need to know more about the sociocultural aspect of VRALL. More specifically, we need to know more about how learners dynamically interact with each other and with their teacher on VR platforms and how VR may influence such interactions. In addition, we need to know more about the specific impacts of individual differences such as gender, age, motivation, and language learning experiences on the implementation of VRALL. Such knowledge may help improve the effectiveness of VRALL in the long run.

In addition, our review calls for more longitudinal studies with a longer duration and experimental studies with delayed post-tests. Longitudinal studies with a longer duration may reveal the more consistent phenomena and the inherent nature of VRALL rather than the ephemeral phenomena during any language learning, which is a risk faced by studies with a short duration. Likewise, studies with delayed post-tests may capture the more stable, long-term effects of VR technology on language learners.

## 5. Conclusion

While reviewing the latest research on VRALL in 2018–2022, we aimed to identify the new trends in research and the benefits and drawbacks of VRALL. We found that the number of research has been rising, especially after the outbreak of the pandemic in 2020, and that the scope of the research has been expanding. Moreover, VR has been proven to be beneficial for language learning in both cognitive and affective aspects despite its drawbacks.

Based on these findings, we put forth some implications for practitioners and researchers. For practitioners, these findings may serve as reference to improve VR language learning platforms and materials to satisfy the various needs of different language learners. For researchers, more future research needs to be done to uncover the general working mechanism and the more specific patterns of VRALL.

It is noteworthy that the empirical studies reviewed here are not exhaustive, as we had selected the studies from major journals and conferences on technology and language learning. There may be related studies in some other journals and databases as well. However, our findings based on these studies are representative enough to update our understanding of VRALL.

## Author contributions

CH came up with the idea of this review article and planned the whole study and did most of the data analysis and wrote the results, discussion, and conclusion sections. CH and JW did data collection and preliminary data analysis together and did the revision and proofreading together. JW wrote the introduction and methodology sections. All authors contributed to the article and approved the submitted version.

## Funding

This research was supported by Shenzhen Technology University Teaching Reform Grant (No. 20221032) and the grant for Center for Research on International Chinese Education 2022–2023 from the Center for Language Education and Cooperation (No. 22YHJD1048).

## Conflict of interest

The authors declare that the research was conducted in the absence of any commercial or financial relationships that could be construed as a potential conflict of interest.

## Publisher’s note

All claims expressed in this article are solely those of the authors and do not necessarily represent those of their affiliated organizations, or those of the publisher, the editors and the reviewers. Any product that may be evaluated in this article, or claim that may be made by its manufacturer, is not guaranteed or endorsed by the publisher.
